# Five-year outcomes of a randomized controlled trial evaluating a non-adjustable ring in Roux-en-Y gastric bypass

**DOI:** 10.1007/s00464-025-11545-3

**Published:** 2025-02-14

**Authors:** Sietske Okkema, Abel Boerboom, Willem den Hengst, Edo Aarts, Frits Berends, Eric Hazebroek

**Affiliations:** 1 Department of Surgery, Rijnstate Hospital, Postal Number 1190, 6800TA Arnhem, The Netherlands; 2 Weight Works Clinics, Amersfoort, the Netherlands; 3 Wageningen University and Research, Wageningen, The Netherlands

**Keywords:** Bariatric surgery, Roux-en-Y gastric bypass, RYGB, Banded gastric bypass, Weight loss, Long-term follow-up

## Abstract

**Background:**

Several retrospective studies suggest that adding a non-adjustable silicone ring to a Roux-en-Y gastric bypass (RYGB) results in more weight loss and prevents weight regain in the long term. The aim of this study was to evaluate the effect of a banded Roux-en-Y gastric bypass (B-RYGB) on weight loss outcomes in a randomized controlled trial (RCT).

**Methods:**

In this single center RCT, 130 patients were divided into two groups: a standard Roux-en-Y gastric bypass (S-RYGB) or a B-RYGB using a Minimizer® ring. Subsequently, weight loss, morbidity, reduction of obesity-associated medical conditions, quality of life (QoL), and complication rates were measured during a follow-up period of five years. A two-sided *p* < 0.05 (with 95% confidence interval) indicated statistical significance.

**Results:**

After five years, mean percentage total body weight loss (%TBWL) was 30.5% in the S-RYGB versus 31.8% in the B-RYGB group (*p* > 0.05). The follow-up percentage was 81%. Overall, no significant differences in complication rates, resolution of obesity-associated medical conditions, and QoL were found between the two groups. In the B-RYGB group, 8 (12%) silicone rings were removed due to symptoms of dysphagia.

**Conclusion:**

B-RYGB is a safe procedure showing similar comorbidity when compared to a S-RYGB. However, B-RYGB led to a higher rate of postoperative dysphagia which poses a risk of ring removal over time. The results from this RCT do not support the hypothesis that implantation of a non-adjustable silicone ring improves long-term weight loss outcomes.

**Graphical abstract:**

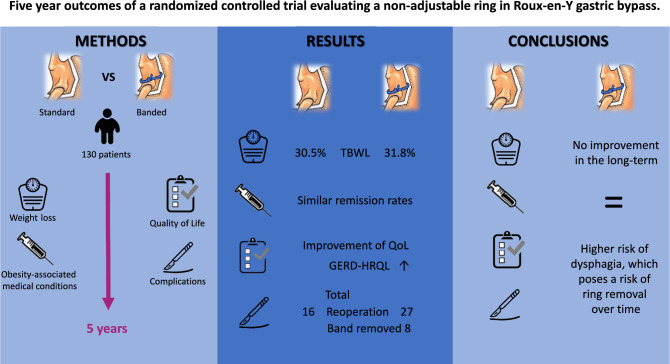

Although the gastric sleeve is currently the most performed procedure worldwide, the Roux-en-Y gastric bypass (RYGB) still holds its ground as one of the most performed metabolic and bariatric procedures [1]. Especially when patients suffer from type 2 diabetes, the RYGB remains the preferred procedure for many surgeons [2]. Remarkably, the basic design of the RYGB has not changed much since its introduction in the 1960s by Mason and Ito [3]. No international standardized protocol on how to perform a RYGB exists, and anatomical and technical aspects of the procedure are often based on local experience.

In search of a standardized design, it is necessary to take a critical look at the various aspects of gastric bypass construction. One of the possible alterations in pouch design is adding a band or ring to the gastric pouch in order to prolong gastric emptying rate and influence satiety.

Placement of an adjustable gastric band has been studied extensively, but is not recommended because of the high number of band related complications [4]. However, many surgeons suggest that using a non-adjustable silastic ring in a primary RYGB results in improved weight loss outcomes with acceptable complication rates [5]. The main beneficial effect lies in the fact that placing the ring proximal to the gastroenterostomy could potentially prevent dilatation of the gastric pouch. Since pouch enlargement is often associated with poorer weight loss and weight regain, prevention of dilatation might improve long-term results after RYGB surgery [6]. However, the majority of metabolic and bariatric surgeons do not perform a banded Roux-en-Y gastric bypass (B-RYGB) due to concerns of postoperative dysphagia and long-term band related complications [7]. Also, adding a ring to the procedure adds to procedure time and increases initial operation costs. There are several studies describing the aforementioned positive effects of a primary B-RYGB, however most of these are observational and descriptive in design [8–13].

Large randomized controlled trials (RCTs) with sufficient length of follow-up are necessary to determine the role of the B-RYGB in the field of metabolic and bariatric surgery (MBS).

## Methods

This study is part of a series of four RCTs investigating possible gripping points for improvement in RYGB design [14–16]. Three studies have been published previously and were designed to investigate different pouch configurations (EXTENDED POUCH trial [14]) and limb length (ELEGANCE and ELEGANCE REDO trials [15, 16]). The present study (BANDOLERA trial) was designed to specifically look at the effect of adding a non-adjustable silastic ring to primary gastric bypass surgery on weight loss, remission of obesity-associated medical conditions, quality of life (QoL), and complication rates.

The protocol of the BANDOLERA trial was approved by the Central Medical Committee for Research in humans and the local committee in our hospital and registered at the clinical registry of clinicaltrials.gov (NCT02545647). The study was designed as a single center, randomized controlled trial conducted following the CONSORT 2010 guidelines and was in accordance with the Declaration of Helsinki.

### Patients

Patient selection for MBS was based on the IFSO criteria (Body Mass Index (BMI) ≥ 35 kg/m^2^ with an obesity-associated medical condition or BMI ≥ 40 kg/m^2^). All patients referred to our center for primary RYGB surgery were assessed for eligibility. Additional exclusion criteria for this study were secondary MBS, any form of inflammatory bowel disease, renal dysfunction (GFR < 30 ml/min/1.73m^2^), and therapy-resistant reflux disease. When patients were willing to participate, the surgeon discussed possible risks and benefits with the patient and afterward handed over an information brochure about the study. Patients had two weeks to consider participation. After these two weeks, written informed consent (two-fold) was obtained from each patient to officially confirm participation.

### Surgical procedures (S-RYGB and B-RYGB)

A standardized laparoscopic technique was used to create an antecolic antegastric RYGB. This technique was similar to the basic technique used in all four randomized trials to be able to compare outcomes [14–16]. All procedures were performed by four experienced surgeons (> 500 RYGB cases before study start). A small gastric pouch of 40–50 ml was constructed using three blue 60 mm linear staplers (Echelon, Ethicon, Johnson & Johnson, New Brunswick, New Jersey, USA) placed against a 40 French gastric tube. An alimentary limb of 150 cm and a biliopancreatic limb of 75 cm were created. The gastroenterostomy and enteroenterostomy were performed using a 35 mm and 60 mm linear stapler (ETS, Ethicon, Johnson & Johnson, New Brunswick, New Jersey, USA), respectively, combined with a barbed suture (V-loc™, Medtronic, Minneapolis, MN, USA). To test the integrity of the anastomosis, an air leak test was performed. Mesenteric defects were closed with a double layer of hernia staples (EMS, Ethicon, Johnson & Johnson, New Brunswick, New Jersey, USA).

**Fig. 1:**
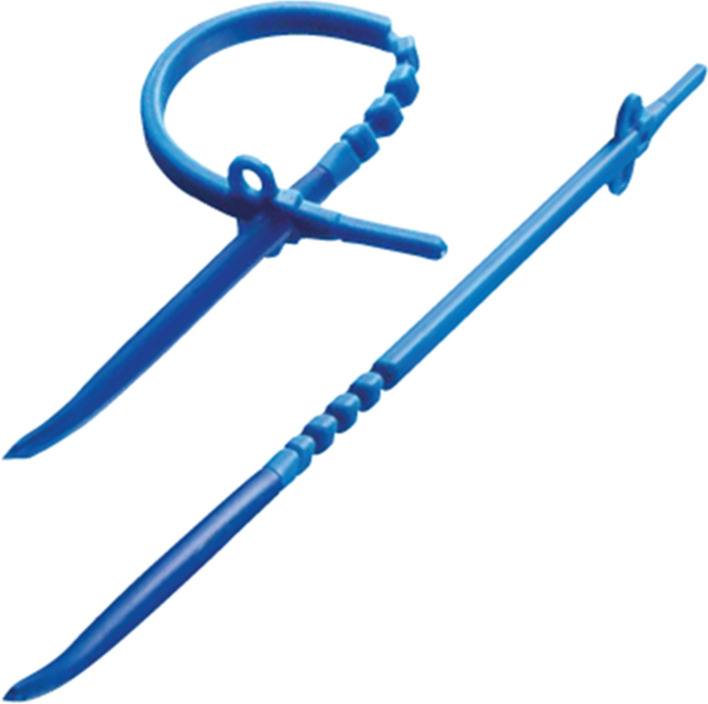
Minimizer ring

The B-RYGB was performed in exactly the same manner. After the air leak test, a perigastric tunnel was created through the omental burse from medial to lateral, positioned two centimeters above the gastroenterostomy. The non-adjustable silicone ring (Minimizer®, Bariatric Solutions, Stein am Rhein, Switzerland) (Fig. 1) was passed through this tunnel from the lateral side. After insertion of a 40 French tube into the pouch, the ring was closed approximately two centimeters above the gastroenterostomy at one of the four closing positions enabling to close the ring at 6.5, 7.0, 7.5, or 8.0 cm circumference. As suggested by the manufacturer, an additional 5 mm instrument should easily pass between the pouch and the ring when closed. The soft needle at the tip of the ring was cut and removed after the ring was fixated using two non-absorbable sutures.

### Primary and secondary outcomes

The primary outcome of this study was weight loss expressed as percentage total body weight loss (%TBWL). Percentage TBWL was defined as weight loss divided by weight before surgery. Weight loss was also calculated and expressed as excess weight loss (%EWL), defined as weight loss divided by excess weight before surgery above a BMI of 25 kg/m^2^. Additionally, weight regain (WR) was defined by weight at the time of follow-up minus weight at nadir, divided by weight at nadir. An increase of more than 15% was indicated as significant [17, 18]. Differences between groups were documented over a period of 5 years.

Secondary outcomes were resolution of the most common obesity-associated medical conditions: type 2 diabetes mellitus (T2DM), hypertension (HT), and dyslipidemia (DL). Additionally, complications after surgery including complaints of reflux disease, nutritional deficiencies, quality of life (QoL), and complaints of dumping were assessed. Obesity-associated medical conditions were defined using the following criteria: for T2DM the use of antidiabetic and/or a fasting glucose > 7 mmol/l and/or a HbA1c ≥ 6.5%, for HT the use of antihypertensive drug therapy and for DL the use of lipid-lowering medication or an elevated level of total cholesterol (> 5.0 mmol/L), elevated level of triglycerides (2.0 > mmol/L) or both [19]. Remission of T2DM was defined as discontinuation of antidiabetic medication for at least one year with normal laboratory values (HbA1c < 6%). Partial remission was defined as sub-diabetic hyperglycemia (HbA1c 6–6.4%) in the absence of antidiabetic medication. Improvement was a reduction of antidiabetic medication and unchanged when no difference to the preoperative situation was documented. Recurrence was defined as a HbA1c ≥ 6.5% or the need for antidiabetic medication after a period of complete or partial remission. Remission of HT was defined as the discontinuation of the antihypertensive medication with a normotensive blood pressure, this was defined as a blood pressure between 100/60 mmHg and 140/90 mmHg [20]. If patients had a decrease in dose or number of antihypertensive medication, the condition improved. Remission of DL was defined as the discontinuation of lipid-lowering medication with a normal lipid panel. Improvement was defined as a decrease in dose or number of medications with equivalent control of dyslipidemia. Nutritional deficiencies were defined as serum levels falling below the lower normal limit. Complaints of gastroesophageal reflux disease (GERD) were assessed using the GERD-Health Related Quality of Life (GERD-HRQL) which contains ten questions concerning reflux and dysphagia. The score of this questionnaire can vary from zero (no complaints) to 50 (very severe complaints). In addition, QoL was assessed using the Bariatric Analysis and Reporting Outcome System (BAROS) and the RAND-36 [21, 22]. At four and five years, dumping related complaints were assessed using the Sigstad and Arts scoring systems, which evaluate dumping symptoms and discriminate between early and late dumping symptoms, respectively [23, 24].

### Perioperative care

Prior to surgery, all patients were screened and underwent extensive multidisciplinary educational lifestyle group sessions with a psychologist and a dietician to prepare them for a change in lifestyle after surgery. These sessions continued after surgery for at least two years. During preoperative consultation at the hospital, patients were screened for nutritional deficiencies and if present these deficiencies were corrected.

Patients were advised to take a lifelong regimen of specialized multivitamin supplements for RYGB patients (FitForMe Forte, Rotterdam, The Netherlands). Additionally, 20 mg of omeprazole for six months, nadroparin 5700 IU for six weeks, and calcium/vitamin D 500 mg/880 IU TID lifelong were prescribed. During the regular annual postoperative medical sessions, which continued up till five years after surgery, patients filled in the questionnaires (GERD-HRQL, Sigstad, Arts, BAROS and RAND-36) and patient weight, medications use, and nutritional status were assessed.

### Sample size, randomization, and blinding

Power analysis led to a sample size of 65 patients per group, based on the assumption that a B-RYGB would lead to 5% higher %TBWL after three years, using a power of 80%, a sensitivity of 95%, a SD of 9.3%, and taken into account a drop-out of 15%.

Randomization was performed by the hospital epidemiologist by applying block randomization with a 1:1 allocation ratio and concealed carrying permuted blocked size of two and four patients. A web-based randomization module was used (Research Manager, Nova Business Software, Zwolle, The Netherlands). Due to the invasive nature of the intervention, patients, surgeons, and researchers could, on practical grounds, not be blinded for group allocation according to the ethics committee. Randomization occurred after patients were sedated, the first day postoperatively patients were informed of the group allocation.

### Statistical methods and monitoring

An independent monitor provided by the local ethical committee of the hospital monitored the study on a regular basis. Deviations (Adverse and Serious Adverse Events) were reported to the Central Medical Committee for Research in humans. Data analysis was performed by the coordinating researcher and the hospital statistician. Per protocol and intention to treat analyses were performed for the primary and secondary outcomes. Protocol violations were excluded for these analyses. The Student *t-*test was used for continuous data and the Fisher’s exact for categorical data. Weight loss during follow-up was analyzed using a mixed-effects model accounting the fixed effects of surgery type (S-RYGB, B-RYGB) and time (T0; T3; T6; T9; T12; T18; T24; T36; T48; T60), and their interaction term, plus random effects of the participants. Time entered the model as a repeated measure using a first-order autoregressive structure with heterogenous variances. Preoperative BMI and age were used as a covariate, and sex and diabetes at baseline were used as a factor, all entering the model as a fixed effect. A two-sided *p* < 0.05 (with 95% confidence interval (CI)) indicated statistical significance. All statistical analysis were performed using IBM SPSS Statistics 25.0 for Windows (IBM Corp., Armonk USA).

## Results

Between August 2015 and February 2016, all 130 patients required for this study were included; 65 patients were enrolled in the S-RYGB group and 65 patients in the B-RYGB group. Baseline patient characteristics between the groups did not differ significantly (Table 1).

**Table 1:** Baseline patient characteristics

	S-RYGB	B-RYGB
Number of patients	65	65
Female (%)	49 (75)	53 (82)
Age, years	45 ± 9	42 ± 9
Length, cm	170 ± 7	170 ± 9
Weight, kg	123 [116–139]	121 [112–137]
BMI, kg/m^2^	42 [40–46]	42 [40–45]

_S-RYGB standard pouch Roux-en-Y gastric bypass, B-RYGB banded Roux-en-Y gastric bypass, BMI body mass index, ±standard deviation_

_No significant differences between the S-RYGB group and the B-RYGB group_

Despite all effort, in total fourteen patients (11%) were lost to follow-up after five years. Eight in the S-RYGB group versus six in the B-RYGB group. Four patients withdrew their consent for participation in the study, three in the S-RYGB groups and one in the B-RYGB group. Two patients deceased during follow-up, one in each group, but not related to surgery. One patient in the S-RYGB group was excluded from the study due to therapy-resistant reflux disease which was missed at the time of randomization. In total, 8 rings were removed during five years of follow-up and in the S-RYGB group one patient received a ring to treat severe dumping syndrome. Over the years, four patients became pregnant, all in the B-RYGB group. Data of patients who had a discontinuation of follow-up were used until the time they were lost to follow-up, withdrew participation, became pregnant or deceased. Data of patients where a ring was removed or placed were used according to the intention-to-treat analysis and remained in their original group of randomization. A follow-up percentage of 92% was achieved after three years, and 81% after five years.

### Weight loss and weight regain

**Fig. 2:**
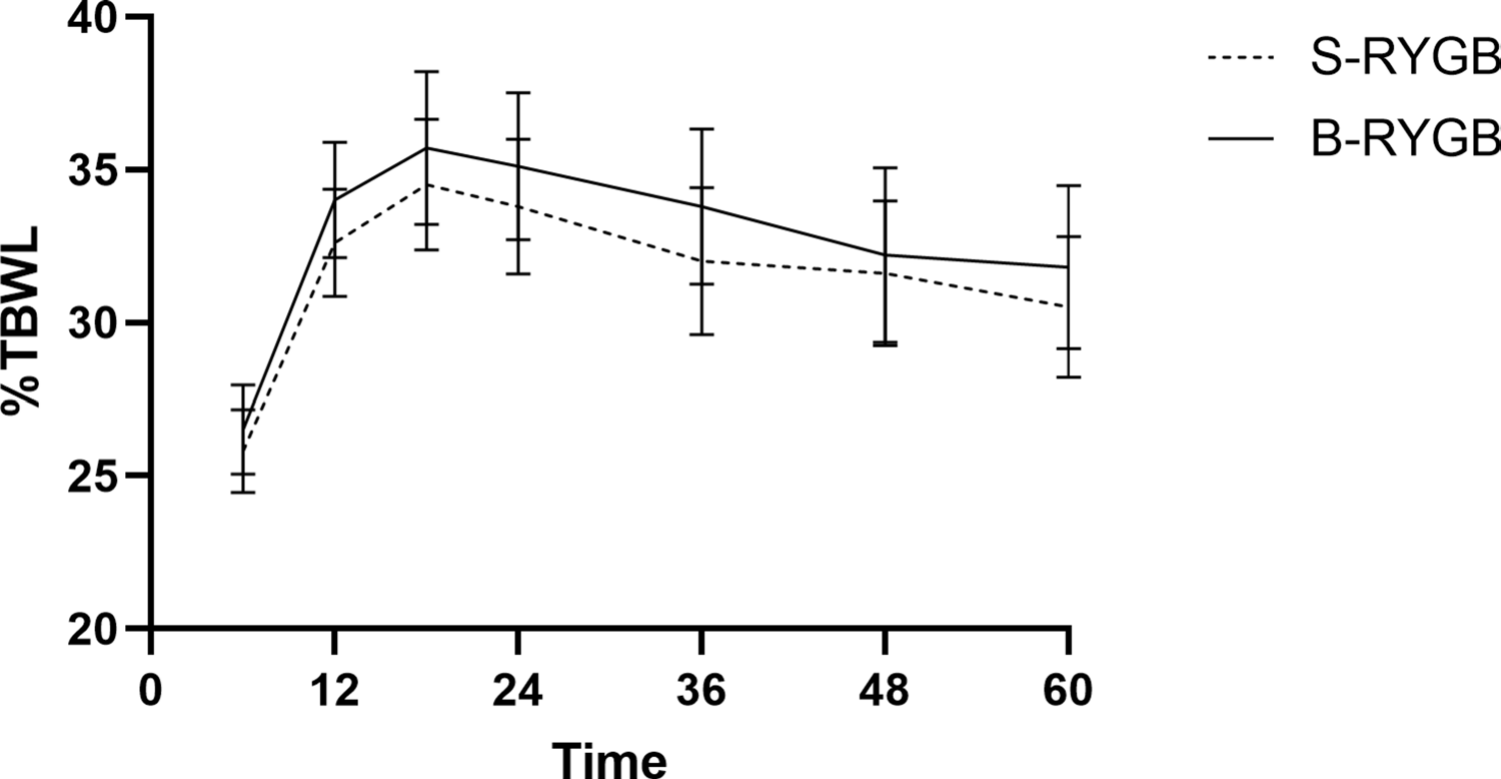
Mean percentage total body weight loss over time of the S-RYGB and B-RYGB. Error bars represent 95% confidence intervals. *S-RYGB* standard Roux-en-Y gastric bypass, *B-RYGB* banded Roux-en-Y gastric bypass

**Fig. 3:**
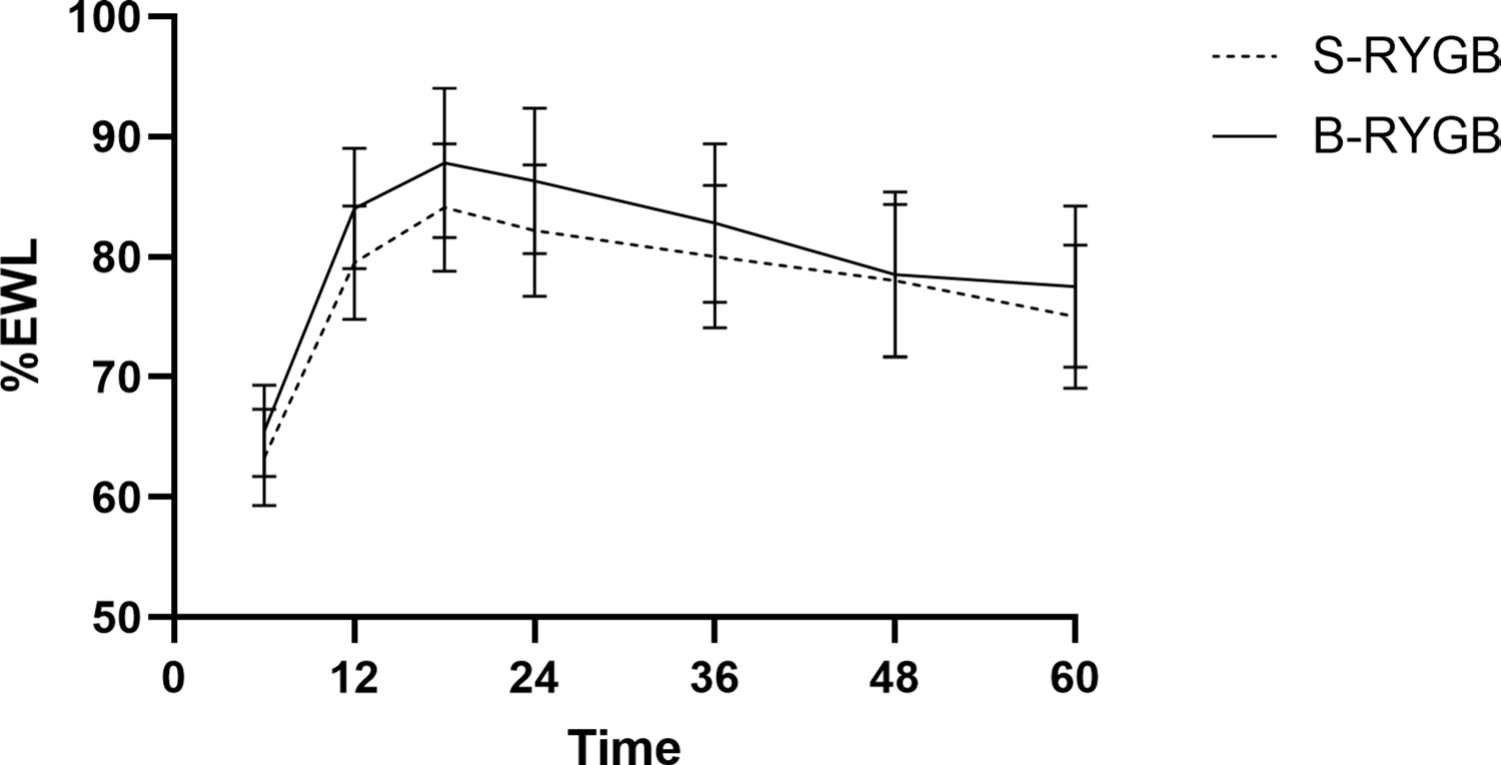
Mean percentage excess weight loss over time of the S-RYGB and B-RYGB. Error bars represent 95% confidence intervals. *S-RYGB* standard Roux-en-Y gastric bypass, *B-RYGB* banded Roux-en-Y gastric bypass

Figures 2 and 3 show weight loss over time between S-RYGB and B-RYGB for %TBWL and %EWL, respectively. After five years, mean %TWBL was 30.5% ± 8.3% in the S-RYGB versus 31.8% ± 9.6% in the B-RYGB group. No significant difference was observed between S-RYGB and B-RYGB in the mixed models effect in terms of %TBWL or %EWL (*p* = 0.684 vs *p* = 0.911 resp.). Although there was a strong effect of time (*p* < 0.001), no strong interaction was observed between time and surgery type (%TBWL *p* = 0.869; %EWL *p* = 0.441), meaning there is no difference in %TBWL or %EWL between S-RYGB and B-RYGB over a period of 6 months to five years.

After three years, three patients in each group suffered from WR (> 15% from nadir weight) with a mean percentage of 17.8% in the S-RYGB versus 20.7% in the B-RYGB group. In one of these patients a ring was removed (WR 22.8%). After five years, seven patients suffered from WR in the S-RYGB, mean WR 20.9%, versus nine in the B-RYGB, mean WR 26.0%. In three patients with WR a ring was removed after five years (mean WR 31.0%). Nine patients reached their lowest weight five years after surgery; 4 S-RYGB versus 5 B-RYGB. No significant differences were found between the groups (3 years *p* = 0.922; 5 years *p* = 0.330).

### Resolution of obesity-associated medical conditions

An overview of the number of patients that achieved improvement and remission of the studied obesity-associated medical conditions can be found in Table 2.

**Table 2:** Remission of obesity-associated medical conditions

		S-RYGB	B-RYGB	*p* value
Type 2 diabetes (%)		18 (28)	9 (14)	0.052
12 months*	Remission	7 (41)	5 (56)	0.810
	Partial Remission	3 (18)	1 (11)	
	Improved	6 (35)	3 (33)	
	Recurrence	–	–	
	Unchanged	1 (6)	–	
	Unknown	–	–	
36 months**	Remission	8 (47)	4 (50)	0.530
	Partial Remission	3 (18)	1 (13)	
	Improved	5 (29)	1 (13)	
	Recurrence	–	1 (13)	
	Unknown	1 (6)	1 (13)	
60 months***	Remission	6 (40)	3 (60)	0.539
	Partial Remission	2 (13)	1 (20)	
	Improved	5 (33)	–	
	Recurrence	1 (7)	1 (20)	
	Unknown	1 (7)	–	
Hypertension (%)		29 (45)	27(42)	0.594
12 months^+^	Remission	6 (25)	4 (25)	0.985
	Improved	14 (58)	9 (56)	
	Unchanged	4 (17)	3 (19)	
	Unknown	–	–	
36 months^++^	Remission	4 (17)	3 (20)	0.249
	Improved	15 (65)	6 (40)	
	Unchanged	2 (9)	1 (7	
	Unknown	2 (9)	5 (33)	
60 months^+++^	Remission	4 (21)	3 (25)	0.598
	Improved	10 (53)	8 (67)	
	Unchanged	3 (16)	1 (8)	
	Unknown	2 (11)	–	
Dyslipidemia (%)		16 (25)	12 (19)	0.298
12 months^#^	Remission	7 (47)	4 (44)	0.407
	Improvement	2 (13)	–	
	Unchanged	6 (40)	4 (44)	
	Unknown	–	1 (11)	
36 months^##^	Remission	4 (31)	1 (13)	0.304
	Improvement	1 (8)	–	
	Unchanged	7 (54)	4 (50)	
	Unknown	1 (8)	3 (38)	
60 months^###^	Remission	3 (27)	2 (29)	0.862
	Improvement	1 (9)	1 (14)	
	Unchanged	6 (55)	4 (57)	
	Unknown	1 (9)	–	

S-RYGB standard pouch Roux-en-Y gastric bypass, B-RYGB banded Roux-en-Y gastric bypass

No significant differences between the S-RYGB group and the B-RYGB group

*Exclusion 1 S-RYGB. **Exclusion 1 S-RYGB vs 1 B-RYGB. ***Exclusion 3 S-RYGB vs 4 B-RYGB

^+^Exclusion 5 S-RYGB vs 11 B-RYGB. ^++^Exclusion 6 S-RYGB vs 12 B-RYGB. ^+++^Exclusion 8 S-RYGB vs 15 B-RYGB

^#^Exclusion 1 S-RYGB vs 3 B-RYGB. ^##^Exclusion 3 S-RYGB vs 4 B-RYGB. ^###^Exclusion 5 S_RYGB vs 5 B-RYGB

#### Type 2 diabetes

At baseline, 27 (21%) patients were diagnosed with T2DM, 18 (28%) in the S-RYGB group and 9 (14%) in the B-RYGB group (*p* = 0.052). Patients with glucose intolerance (a fasting glucose > 7 mmol/l and/or a HbA1c ≥ 6.5% without using antidiabetic medication) were present in both groups, two in the S-RYGB group and two in the B-RYGB group. No significant differences between groups were found in remission or improvement rate.

#### Hypertension

In the S-RYGB group 29 (45%) patients suffered from hypertension at baseline versus 27 (42%) in the B-RYGB group. Remission and improvement rates were comparable in both groups, no significant differences were found.

#### Dyslipidemia

The number of patients that suffered from dyslipidemia, with or without lipid-lowering medication, at baseline was 28 (22%), 16 (25%) in the S-RYGB group and 12 (19%) B-RYGB group. No significant differences were found in remission or improvement rate between groups.

#### Complications

All short- and long-term complications are listed in Table 3. In total, 15 (12%) patients had a short term complication after surgery. In the B-RYGB group two rings were removed within two weeks after surgery, both due to complaints of dysphagia. In one of these patients a gastroscopy showed a stenosis based on an ulcer at the gastroenterostomy and in the other patient a barium swallow imaging study showed slow passage at the gastroenterostomy. Both rings were removed and eventually the complaints of dysphagia disappeared. In the B-RYGB group one patient required a cholecystectomy.

**Table 3:** Short- and long-term complications at 5 years follow-up

	S-RYGB	B-RYGB	*p* value
Short term (< 30 days)			
Total number of patients (%)	7 (11)	8 (12)	0.784
Reoperation	2	4	
Ring removal	–	2	
Blow-out stomach remnant	1	–	
Bleeding	–	1	
Cholecystectomy	1	1	
Conservatively treated bleeding	2	3	
Pulmonary embolism	1	–	
Readmission	2	1	
Mortality	–	–	
Long term (> 30 days)			
Total number of patients (%)	16 (25)	22 (34)	0.270
Reoperation	14	23	
Ring removal	–	6	
Cholecystectomy	5	6	
Internal herniation	5	4	
Revision gastroenterostomy	1	1	
Ring placed	1	–	
Incarcerated umbilical hernia	–	1	
Diagnostic laparoscopy	2	5	
Gastric ulcer	1	2	
Dysphagia	1	3	
ACNES*	1	–	
Readmission	2	3	
Mortality	1	1	

_S-RYGB standard pouch Roux-en-Y gastric bypass, B-RYGB banded pouch Roux-en-Y gastric bypass_

_No significant differences between the S-RYGB group and the B-RYGB group_

_*Abdominal cutaneous nerve entrapment syndrome_

In the long term, in six patients the ring was removed. In all six patients because of a combination of abdominal pain and persistent dysphagia which could not be explained otherwise. Postoperatively, the complaints of dysphagia disappeared. In one patient in the S-RYGB group a minimizer was placed due to severe dumping syndrome. Two patients died during follow-up, one patient in the B-RYGB group (without abdominal complaints) committed suicide, the cause of death of the patient in the S-RYGB group was unclear and the family did not wish to clarify. No significant differences in short- and long-term complications between the groups were found.

### Nutritional status and compliance

The number of patients that were using the prescribed specialized multivitamin supplements for RYGB patients dropped from 83% after one year to 72% after five years of follow-up. Table 4 gives an overview of the nutritional deficiencies during follow-up. The largest difference between groups was seen in folic acid deficiency at one and two years, but no significant differences were found between groups.

**Table 4:** Nutritional and vitamin deficiencies

	S-RYGB							B-RYGB					
	Baseline (%)	12 m (%)	24 m (%)	36 m (%)	48 m (%)	60 m (%)		Baseline (%)	12 m (%)	24 m (%)	36 m (%)	48 m (%)	60 m (%)
Anemia	3	8	7	6	9	-		2	5	7	6	19	7
Folic acid	22	7	9	15	6	14		27	17	21	20	9	22
Vitamin B_12_	8	–	2	2	-	3		9	2	4	–	–	9
Ferritin	2	–	2	9	9	7		–	–	4	6	16	10
Vitamin D	65	2	7	13	20	17		55	5	12	26	16	15

_S-RYGB standard pouch Roux-en-Y gastric bypass, B-RYGB banded pouch Roux-en-Y gastric bypass_

_No significant differences between groups_

### Quality of life

The mean total GERD-HRQL score after three years was 2.0 in the S-RYGB group and 4.7 in the B-RYGB group (*p* = 0.012). After five years, the score in the S-RYGB group increased to 5.2 and the score in the B-RYGB group showed a decrease to 3.9. Twenty-five percent of patients used a proton pomp inhibitor (PPI) in the S-RYGB group after three years, this percentage remained nearly the same after five years; 26%. In the B-RYGB group, 29% used a PPI after three years which increased to 33% after five years. No significant differences were found between groups.

In the BAROS, after the first year of follow-up 94.3% of all patients scored ‘good’ or better than good with a mean total score of 5.0 in the S-RYGB group versus 4.9 in the B-RYGB group. Up until five years, there was an increase in mean total score to 5.2 in the S-RYGB group versus 5.4 in the B-RYGB group. However, the percentage of patients scored ‘good’ or better than good dropped to 80.8% at this timepoint.

The RAND-36 showed a significant improvement in six of the nine domains; social functioning, emotional well-being, and energy/fatigue showed no significant improvement after 5 years.

Between groups, a significant difference was seen in the domain role limitations due to emotional problems after three years. This difference disappeared after 5 years, no significant difference was found at this timepoint between groups.

At four and five years, an indication of dumping related complaints was made using the Sigstad and Arts questionnaire. A score above seven indicates dumping in the Sigstad questionnaire, at both timepoints the mean scores of the groups was beneath this cut-off point. No significant differences were found between the S-RYGB and B-RYGB group at four and five years (*p* = 0.079 and *p* = 0.515, respectively). In patients with dumping based on the Sigstad questionnaire, the Arts questionnaire showed a borderline significant difference between groups in early dumping symptoms (*p* = 0.048) at 5 years in favor of the S-RYGB. One patient suffered from severe complaints after a ring was removed.

## Discussion

The majority of all patients with obesity reach a %TBWL > 25 or %EWL > 50 after surgery, which are the thresholds that are often used to indicate good weight loss [25]. Weight loss above these thresholds is associated with a higher patient satisfactory and increased resolution of obesity-associated medical problems. Unfortunately, weight regain is seen in a subset of patients. Due to the different definitions used in previous studies, the number of patients with weight regain ranges from approximately 16 to 87% [17, 18]. With the definition of weight regain being more than 15% increase to nadir weight, the study of Voorwinde et al. showed that 24% of patients suffer from weight regain [17] and the study of Elhag et al. showed a proportion of 30% of patients [18]. Several factors such as lifestyle, genetic, and technical aspects are thought to have an effect on weight regain after MBS [25]. It must be stressed that weight regain is a physical and psychological burden for patients that weighs heavily on quality of life. Any intervention that could help to prevent weight regain should therefore be welcomed.

Although hard evidence is lacking, it is suggested that placement of a non-adjustable silicone ring could counteract dilatation of the pouch, may delay food passage and pouch emptying and therefore prevent weight regain in RYGB patients [6, 26, 27].

However, the results of our RCT do not support this hypothesis as only a small, non-significant, difference in percentage weight loss is observed in favor of the B-RYGB (%TBWL 31.8 vs 30.5 and %EWL 77.5 vs 75.0). Compared to the literature and previous RCTs conducted in our center in which a gastric bypass with 75 cm biliopancreatic and 150 cm alimentary limb was used, both the S-RYGB and B-RYGB in this study was associated with %TBWL > 30% during the complete follow-up of the study. These exceptionally good results are worth mentioning as the literature shows a %TBWL of approximately 27% after RYGB in the long term [12, 14, 16]. Besides weight loss resulting from the initial procedure, we also could not demonstrate a significant difference in recurrent weight gain after three and five years between the two study groups.

The idea of preventing weight regain by preventing dilatation of the pouch is not new. Several adjustable and non-adjustable prosthesis, (self-constructed) devices and materials to reinforce the gastric pouch and prevent enlargement have been used and investigated, but so far, no definite conclusions could be drawn from these studies [5, 7].

It should be acknowledged that any surgical procedure using implantation of a foreign body may hamper clinical outcomes in terms of safety. The same applies to the safety of a non-adjustable (silicone) gastric ring. The number of ring removals in literature ranges from 0 to 21.7% [7, 10, 12, 13, 28, 29]. In the present study, the percentage of ring removals was 12%, all due to disabling dysphagia. As also described in earlier studies, placement of a ring around the pouch is often followed by a higher prevalence of postoperative dysphagia or food intolerance. When considering the use of a non-adjustable ring, surgeons should therefore keep in mind not to close the ring too tight. As advised by the manufacturer, a 40 French stomach tube inside the pouch and an additional 5 mm instrument should easily pass between the pouch and the ring when closed. Increasing the diameter from 5.5 to 6.5 cm led to a balance between weight loss and likelihood to remove the ring due to complaints [6, 30]. The mean circumference of the removed rings in this study was 7.25 cm, which was practically the same as the overall mean circumference of 7.24 cm and considered loose comparing to other studies. This greater circumference could also be an explanation why, in contrast to previous literature, our banded procedures did not show higher weight loss results than the non-banded control group. However, it must be noted that studies on banded procedures should be interpreted with some caution due to differences in design (i.e., randomized or non-randomized/with or without control group) and length of follow-up [8–11].

At the start of this study, this non-adjustable ring was a relatively new device. As was suggested in a multicenter cohort study where a non-adjustable ring was used as a revisional procedure [27], perigastric placement of a non-adjustable ring is associated with a significant learning curve and could therefore partly explain the relatively high number of removals.

Patients in the B-RYGB reported more complaints of heartburn and regurgitation on the GERD-HRQL, which resulted in a higher score and a significant difference between groups at three years. Patients may consider some degree of dysphagia to be a desirable effect of the B-RYGB and subsequently adjust their eating patterns. The altered eating pattern may have a good result in terms of weight loss, but could also be insufficient with a risk of developing nutritional deficiencies. In future studies, dysphagia, its effect on eating behavior and additional information of the eating pattern should be taken into account.

Increased weight loss after MBS is associated with improved resolution of obesity-associated medical conditions. At baseline, the incidence of T2DM in S-RYGB (28%) was two times higher compared to the B-RYGB (14%), approaching significance. Although not significant, this could have confounded the analyses on remission rates of T2DM and therefore no hard conclusions can be drawn from these results.

One of the major strengths of the present study is obviously its randomized design. However, patients were not blinded for treatment. In retrospect, the sample size can be considered a limitation. The drop-out ratio after three years (8%) was within the anticipated ratio of 15%. Although the number of patients who were lost to follow-up increased to 19% after five years, the expected ratio was not calculated for five years after surgery.

Another limitation is the lack of dietary assessment and physical exercise. Due to the randomized design, it can be assumed that both study groups had comparable diets and levels of physical activity based on the perioperative multidisciplinary support program. However, this was not taken into account in the analyses of weight loss and weight regain.

This study is the fourth RCT performed in our center which looked into possible gripping points for improvement of the RYGB design including limb length and pouch size [14, 16]. Because of the reported improvement in short- to mid-term weight loss outcomes in these studies, we also wanted to evaluate a combination of all aforementioned RCTs with a larger sample size. Therefore, we started the UPGRADE study, which is a 3-armed multicenter blinded RCT comparing a standard gastric bypass with an extended pouch gastric bypass and a banded-extended gastric bypass (ClinicalTrials.gov number NCT05357807).

## Conclusion

B-RYGB is a safe procedure showing similar comorbidity when compared to a S-RYGB. However, B-RYGB led to a higher rate of postoperative dysphagia which poses a risk of ring removal over time. The results from this RCT do not support the hypothesis that implantation of a non-adjustable silicone ring improves long-term weight loss outcomes.
